# Supplementary material for: Human click-based echolocation: Effects of blindness and age, and real-life implications in a 10-week training program
Source: PLoS One. 2021 Jun 2;16(6):e0252330. doi: 10.1371/journal.pone.0252330 (PMC8171922; doi:10.1371/journal.pone.0252330)
Supplement: S1 File — (DOCX) [file pone.0252330.s003.docx]

Human click-based echolocation: Effects of blindness and age, and real-life implications in a 10-week training program

**SUPPLEMENTARY MATERIALS**

L. J. Norman^1&^, C. Dodsworth^1^*,* D. Foresteire^1^*,* L. Thaler^1&*^

*^1^ Department of Psychology, Durham University, Durham DH1 3LE, UK*

^&^ joint first authorship

*Corresponding author:

Lore Thaler

[lore.thaler@durham.ac.uk](mailto:lore.thaler@durham.ac.uk)

Department of Psychology, Durham University

Science Site, South Road

Durham DH1 3LE

United Kingdom

<http://orcid.org/0000-0001-6267-3129>

**Methods**

Detection of Sound Frequency Modulation (DFM)

The description provided here is from Thaler & Foresteire (2017). On each trial participants were presented with a pair of tones and they pressed a button whenever they detected a change in frequency in the second tone of a pair. Each pair consisted of a 2-second steady pure tone (incl. 80 ms linear on and off ramp), followed by another 2-second tone (incl. 80 ms linear on and off ramp), that could be either another steady pure tone (‘catch-trial’), or a frequency modulated tone. There was a 300 ms silent gap in between the two tones. The frequency modulation was 300 ms long. To avoid participants predicting when a frequency modulation would occur, the onset time of the modulations was randomized, with the limitation that modulations could only occur after an 800 ms lead-in of the continuous tone. Participants were informed that the first tone was always a steady tone, and they were told that they could use this as a reference for assessing any changes in the second tone. We used three increments of frequency modulation (0.6, 0.4, 0.2 percent modulation) and 18 tests were presented for each increment. Tests were conducted at centre frequencies of 500 and 2000 Hz. Test trials were preceded by a practice series with increments in frequency modulation that gradually decreased from 5 to 2 percent. Following Carlson-Smith & Wiener (1996), the test was presented at 500 Hz and 2000 Hz at 30 dB HT (as determined for our set-up; a single HT value was obtained for each participant by averaging HT across the right and left ears). Catch trials were used to calculate proportion of false alarms. For each test and participant we then subtracted proportion of false alarms from proportion of correct detections. For one participant who was sighted (aged 25 yrs) an error occurred and data was not saved.

Detection of Change in Sound Intensity (DCI)

The description provided here is from Thaler & Foresteire (2017). We used three increments of intensity modulation (1.2, 1.0 and 0.8 dB) and 18 tests were presented for each increment. There were also 18 catch trials. Tests were conducted at centre frequencies of 500 and 2000 Hz. Participants were instructed to press a button whenever they detected a jump in loudness. Test trials were preceded by a practice series with increments in intensity that gradually decreased from 5 dB to 2 dB. Following Carlson-Smith & Wiener (1996), the test was presented at 500 and 2000 Hz at 45 and 35 dB Hearing Threshold (HT), respectively (as determined for our set-up; a single HT value was obtained for each participant by averaging HT across the right and left ears). Catch trials were used to calculate proportion of false alarms. For each test and participant we then subtracted proportion of false alarms from proportion of correct detections. For one participant who was sighted (aged 25 yrs) an error occurred and data was not saved.

**Results**

Size task - Results from Non-parametric tests on distance data

In addition to the parametric tests reported in the main text, we analyzed data using non-parametric tests because distance could only increase, once someone had improved their distance it could not decrease again. Consistent with main effect of session in the ANOVA, a non-parametric Friedman’s test for the effect of session applied to combined data from SCs and BCs was also significant (X^2^(N=26; df=19)=141.662; p<.001), and was also significant separately for SCs (X^2^(N=14; df=19) = 102.581; p<.001) and BCs X^2^(N=12; df=19)=40.923;p=.002). Focussing on performance in session 20, a non-parametric Mann-Whitney-U test (U=63; z=-1.249; p=.212) showed no significant difference between BC and SC groups. This further supports the conclusion that, as training progressed, participants were able to perform the task at farther distances, i.e., their performance improved, and this seemed to be the same for both blind and sighted participants.

Orientation task – Results from non-parametric tests on distance data

Consistent with main effect of session in the ANOVA, a non-parametric Friedman’s test for the effect of session applied to combined data from SCs and BCs was also significant (X^2^(N=26; df=19)=166.193; p<.001), and it was also significant separately for SCs (X^2^(N=14; df=19) = 100.909; p<.001) and BCs (X^2^(N=12; df=19)=68.456;p<.001). Focussing on performance in session 20, a non-parametric Mann-Whitney-U test (U=75.5; z=-.481; p=.630) showed no significant difference between BC and SC groups. This further supports the conclusion that, as training progressed, participants were able to perform the task at farther distances, i.e., their performance improved, and this seemed to be the same for both blind and sighted participants.

Virtual Navigation Task – Results from Analyses comparing sessions 14 and 15

To determine whether the randomised starts and error timeouts did in fact lead to greater difficulty, we compared performance between sessions 14 and 15 using a mixed model ANOVA with session (14, 15) and group (blind, sighted) as factors.

Time taken to complete Mazes

For sessions 14 vs. 15, minimum effect size for the main effect of ‘session’ and the interaction effect was .262, and for the main effect of ‘group’ it was .247. Comparison of time taken to complete mazes between sessions 14 and 15 showed a significant effect of session (F(1,24)=113.091; p<.001; η^2^_p_: .825), a significant effect of group (F(1,24)=9.659; p=.005; η^2^_p_: .287), as well as a significant interaction between session and group (F(1,24)=5.994; p=.022; η^2^_p_: .200). We followed up the significant interaction with t-tests. First, paired t-tests showed a significant increase in completion time from session 14 to 15 for both groups (SC: t(13)=5.858; p<.001; mean diff: 48.43; SD: 30.93; BC: t(11)=9.235; p<.001; mean diff: 77.39; SD: 29.03). This is also visible in Figure 7, and is likely due to the error timeouts and randomised starting orientations that were introduced from session 15 onwards, which would impose additional delays and possibly a more careful strategy when completing the task. Importantly, however, independent samples t-tests showed that, whilst in session 14 there was no significant difference between participant groups (t(24)=1.502; p=.146), in session 15 SCs completed the mazes on average in less time than BCs (t(24)=3.723; p=.001). Figure shows these data.

In sum, when error timeouts and random starting orientations were introduced from session 15 onwards, SCs were less affected (i.e. they slowed down less) than BCs, but this could be explained by the fact that for session 15-20 older age was associated with longer completion times, and our group of blind participants was older.

Number of Collisions

For sessions 14 vs. 15, the minimum effect size for the main effect of ‘session’ and the interaction effect was .262, and for the main effect of ‘group’ it was .247. Comparison of number of collisions between sessions 14 and 15 showed that whilst SCs had significantly fewer collisions (mean: 2.018; SD: 1.89) than BCs (mean: 4.597; SD: 1.89; F(1,25)=12.000; p=.002; η^2^_p_: .333), there was no effect of session (F(1,24)=4.000; p=.057; η^2^_p_: .143), and also no interaction between session and group (F(1,24)=3.572; p=.071; η^2^_p_: .13). This suggests that the error timeouts and random starting orientations introduced in session 15 did not affect the number of collisions.

In sum, error timeouts and random starting orientations introduced from session 15 onwards did not have any effect on number of collisions.

Proportion of Successful Maze Completions

For sessions 14 vs. 15, minimum effect size for the main effect of ‘session’ and the interaction effect was .262, and for the main effect of ‘group’ it was .247. Comparison of proportion of successes between sessions 14 and 15 showed a significant effect of session (F(1,24)=38.445; p<.001; η^2^_p_: .616), a significant effect of group (F(1,24)=13.925;p=.001; η^2^_p_: .367), as well as a significant interaction between session and group (F(1,24)=7.116; p=.013; η^2^_p_: .229). We followed up the significant interaction with t-tests. First, using paired t-tests to compare performance between session 14 and 15 for each group separately showed a significant drop in proportion of successful maze completions for both groups (sighted: t(13)=3.161; p=.008; mean diff: .131; SD: .155; blind: t(11)=5.140; p<.001; mean diff: .329; SD: .222). This is also visible in Figure 9, and is likely due to the error timeouts and unpredictable starting orientations that were introduced from session 15 onwards, which could possibly make it more difficult to complete the mazes within the time limit. Importantly, however, independent samples t-tests comparing performance between groups separately for session 14 and 15, showed that whilst in session 14 there was no significant difference between participant groups (t(12.839)=2.207; p=.064), in session 15, SCs completed more mazes (mean: .85, SD: .17) successfully than BCs (mean: .56; SD: .23) (t(24)=3.681; p=.001). Figure 9 illustrates this, and suggests that participants who were sighted were less affected by the additional timeouts and random starting orientations as compared to participants who were blind.

In sum, when error timeouts and random starting orientations were introduced from session 15 onwards, SCs were less affected than BCs, but this could be explained by the fact that older age is associated with fewer successful maze completions, and our group of BCs was older.

**References**

Carlsson–Smith C, Wiener WR. The auditory skills necessary for echolocation: A new explanation. Journal of Visual Impairment Blindness. 1996; 90: 21-35.

Thaler L, Foresteire D. Visual sensory stimulation interferes with people’s ability to echolocate object size. Sci Rep. 2017; 7: 1-10.
