# Supplementary material for: Human click-based echolocation: Effects of blindness and age, and real-life implications in a 10-week training program
Source: PLoS One. 2021 Jun 2;16(6):e0252330. doi: 10.1371/journal.pone.0252330 (PMC8171922; doi:10.1371/journal.pone.0252330)
Supplement: Attachment — Submitted filename: resp_to_reviews_17feb2021 - Copy.docx [file pone.0252330.s004.docx]

Dear Professor Ptito,

We thank the reviewers for their comprehensive and constructive feedback.

We have revised our manuscript following their suggestions, and believe that it is improved as a result.

Here is a summary of the major changes that we have made:

- We have removed various experiments & results from the manuscript (i.e. generalization tasks as suggested by reviewer 2). This shortened the manuscript and improved clarity and coherence
- We have moved descriptions of DCI and DFM hearing tests to a supplementary materials file
- We have moved descriptions of results comparing performance in the virtual navigation task across session 14 and 15 to a supplementary file. These analyses were not central to the arguments made in the manuscript; they were merely used to determine the effect of making the virtual navigation task more difficult by introducing random starts and timeouts
- We have included non-parametric analyses for our measure of performance based on stimulus distance; these results are in agreement with results from parametric analyses, and for that reason we have supplied them in supplementary materials (with reference to them and their implications in the main text). We are happy to include them in the main text if the reviewers prefer.
- We have added non-parametric tests for all comparisons between experts and trained participants, keeping sighted and blind groups separate and treating experts as a group; accordingly, we have removed individual comparisons via t-tests
- We have removed distance comparisons to experts
- Throughout the manuscript conclusions etc have been revised
- We have reduced repetition and redundancy in the writing wherever possible in order to reduce the length of the manuscript

These changes have resulted in the length of the manuscript to be reduced by 2,500 words. We have made various other changes and detailed them below in the responses to reviews. Our responses are in red font, the reviewers’ comments in black font.

With best wishes, Lore Thaler

Reviewer #1: The manuscript “Human echolocation: Effects of blindness and age and real-life implications in a 10- week training program” details a training program for teaching humans click-echolocation. It includes groups of 14 sighted and 12 blind participants who undergo echolocation training and an additional 6 participants who are expert echolocators. The authors find that participants significantly improved over time in a number of different spatial auditory tasks and most importantly that it significantly improved the life of the blind participants several months post training.

Helping humans who are blind is a critical challenge, and echolocation has long been considered to hold great potential for this endeavor. The current manuscript is a significant contribution to this effort as it is one of the first demonstrations of long term benefits (the survey results alone could have held up the paper), and adds a bunch of interesting specific findings along the way.

On the other hand, the manuscript currently has significant statistical issues in many of it’s analyses, which have significant implications for many small results, that simply must be corrected before I could recommend it for publication.
Note though, that while they have significant implications for several of the specifics findings (especially regarding comparisons across groups), they do not affect the manuscripts key results and thus i still see great potential for this manuscript to be corrected.

I encourage authors to correct these statistical issues and side-results and improve this important work.

We thank the reviewer for their feedback and have taken their comments on board for our revision.

In further details:
===========
1. Statistics
There are a number of statistical issues with this manuscript, but I think they are most exemplified by the contrast between the figures and the calculations in the text. Can one really look at figures 6-9 and say that there is no difference between the sighted and blind groups? And no difference between them and the experts perform within their range?
I think the problem lays in several problematic uses of statistics. These include:

a. Confusing discrete and continuous variables analysis - the distance from the target in the first two tasks was discrete, with a series of potential distances with a fixed distance apart. This requires discrete analysis tools rather than continuous ones - for example a mean value somewhere between the discrete values is not meaningful. Therefore for example, it is the usual practice to use Median values rather than Means. Another example is the recommended use in these cases of non parametric statistical tests, such as rank tests for this kind of data. Using these tools on figures 6 and 7 leads to the result obvious to the viewer whereby the experts are significantly better (as their ranks are maximal) and that the sighted develop a small but clear advantage over the blind group.

In response to reviewer’s suggestion we now use non-parametric tests to compare data from experts to data from trained participants. We also provide non-parametric analyses for the distance variable. Results for the distances using non-parametric tests are in agreement with results from parametric analyses. Thus, for brevity, we have supplied the non-parametric statistics in supplementary materials (with reference to them in the main text where relevant). We are happy to move them into the main text if this is preferred.

Regarding the discrete vs. continuous nature of the distance variable:

The steps in between levels are metric & equal, so that our distance measure qualifies as data suitable to analyse with ANOVA (i.e. interval/ratio). We agree that using 33cm steps instead of 1cm steps for example seems ‘discrete’, but it is a matter of scale. As mentioned above, however, we now also provide non-parametric statistics in the supplementary materials.

b. Joining the results from the blind and sighted groups in the final session - authors show that that there is no effect of group on the overall level, and use this as justification to join the blind and sighted groups to one in the final trial for comparison with experts. This is not legitimate. To do so, they would need to directly compare session 20, and if they found no difference specifically there, it would be ok to merge the groups for comparison with the experts. Given the results in the figures, I highly doubt that there is no significant, or at least close to significant, difference between the groups in that trial. Merging these groups on that trial despite their differences leads to a much higher variance, which in turn is large enough to reach the experts scores.

Reviewer 2 also commented on this issue. We now perform separate comparison of experts to sighted and blind participants.

Page 22 (line 439) – size task

“Focusing only on performance in the final session, SCs performed better than BCs (t(24)=2.417; p=.024). Furthermore, EEs (n=3) performed significantly better than SCs (U=5; z=-2.042; p=.041) and BCs (U=2; z=-2.311; p=.021) did in the final session.”

Page 23 (line 480) – orientation task

“Furthermore, EEs (n=5) did not perform significantly better than either BCs (U=12; z=-1.901; p=.057) or SCs (U=21; z=-1.310; p=.190) did in the final session.”

Page 25, (line 531) – maze time

“Focusing only on completion time in session 14, there was no significant difference between SCs and BCs (t(24)=1.502; p=.146). Furthermore, BCs did not significantly differ in completion time compared to EEs (n=4; U=17; z=-.849; p=.396), but SCs were significantly faster than EEs (U=9; z=-2.018; p=.044).”

Page 27 (line 576) – maze collisions

“Focussing just on number of collisions in session 14, SCs performed significantly better than BCs (t(15.836)=3.006; p=.008), consistent with the significant main effect of ‘group’. Furthermore, EEs (n=4) did not differ significantly compared to either BCs (U=11; z=-1.578; p=.115) or SCs (U=12; z=-1.701; p=.089), suggesting that participants had learned to perform like experts by session 14.”

Page 29 (line 611) – maze success

“Focusing only on performance in session 14, there was no significant difference between BCs and SCs (t(12.839)=2.207; p=.064). Focussing just on session 14, EEs (n=4) did not perform significantly differently compared to either BCs (U=22.5; z=-.187; p=.851) or SCs (U=13.5; z=-1.836; p=.066).”

c. I am not happy about the way the authors treat the distances.
The most basic step of designing paradigms is making sure they differ only in a minimal number of things. This added parameter which is completely different fo the experts and subjects, which changes in a monotonous and discrete manner and which should directly effect difficulty and therefore result success level is very problematic, and especially when mixed into the general statistics.
If I understand correctly distances only improved and not potentially decreased if subjects were not good at the new level? This creates a structure in the trial levels which violates observation independence, which gets even more complicated when this measure is mixed in with others inside the Anovas.
I understand the authors comments about this being even more strict, since in higher distances the task is harder, but it s still a significant change in the subjects task which complicates the choice of statistical tools. I would prefer authors avoid anovas on this, and especially avoid mixing it in with other parameters.
Finally, given the inherent difference in distance to the echolocator group (even ignoring the other statistical issues with this comparison mentioned above), I do not think the result of no difference between them and the other groups can be legitimately supported, and these statements should be removed.

Re distances and analysing the effect of session (where distances could increase when people improved):

Yes, distances could not decrease, but importantly, for that aspect of data (i.e. effect of session) we used repeated measures ANOVA which does not assume independence of observations across levels. Furthermore, we are careful to look at correlations amongst levels of this variable (i.e. at sphericity) and if there is a concern we used ANOVA that have been corrected for violation of sphericity. Furthermore, the steps in between levels of our distance variable are metric & equal so that our distance measure qualifies as data suitable to analyse with ANOVA.

We understand the concern raised, however. Therefore, if we could we would like to use a non-parametric alternative to mixed ANOVA but we are not aware of appropriate NP procedures. Thus, for analysis of distance for sighted and blind controls we have kept the ANOVAs. But, based on the reviewer’s suggestion we now supplement this with a non-parametric analysis of session (for the distance measure) separately for blind and sighted participants as well as for both groups combined. Results for the distances using non-parametric tests are in agreement with results from parametric analyses. Thus, for brevity, we have supplied the non-parametric statistics in supplementary materials. We are happy to move them into the main text if this is preferred.

Re how to compare experts and trained participants with regard to distances:

Both reviewer 1 and reviewer 2 raised the issue regarding comparison of distance between controls and EEs, and thus we have removed statistical comparison between EEs and controls for the distances.

d. the author compare the experts to the group as a single sample compared to a distribution. This makes sense when there is 1 expert and a large group of subjects. This is much less legitimate when there are 6 experts. Using a non parametric test, and comparing specifically to either of the groups separately (as suggested above) will clearly put most experts way above the others. (except in the computer task, where I think computer skills/litercy might be havig an effect on some experts? Or maybe the difference between virtual echolocation and real world echolocation hinders their habits and therefore performance?). But even if you want to keep the current stats, you need to correct for the number of experts - the chance of getting one expert above the group might be likely for the participants scores distribution, but getting 3 samples all highly above the group mean has a totally different level of significance.
This is especially evident in figure 13b-d here the experts distribution is clearly superior.

Yes we have revised our analysis and now use non-parametric tests treating the echolocators as a group. We have modified our conclusions as appropriate. The results shown in Figure 13 have been removed from the manuscript as per reviewer 2’s suggestion.

Please see our response to earlier comment.

e. the age analysis is significantly complicated by the inherent difference in ages between the groups. If the single elderly subject is removed from the sighted group as an outlier for age, I think a lot of their results on age fall apart. Authors acknowledge their limitations and try to address this, but it is still a significant issue - especially since authors collapse across groups for some analysis (e.g. figures 14, 15) where it seems that trends can be seen for one group and not the other (given that my previous comments might prevent the ability to collapse across groups, authors might need to run al of these separately anyway).

We have now included analyses for age for each group also and show correlations for each group in each scatterplot. For active tasks there are no significant correlations also when these are broken down by group. For the computer based tasks it seems that correlations are generally stronger for the sighted group, but the sign of the relationship is the same in the two groups. We also confirmed that any of our results do not hinge on inclusion/exclusion of particular participants. Removing participants obviously affects statistical power. There was no reason (statistical or otherwise) to be concerned about the oldest subject from the sighted group.

Page 32 (line 685):

“For practical size and orientation tasks, participant improvement was not associated with age or blindness or with any of our hearing measures (max. r value = .373 for the whole group and max r value = -.482 for separate group analyses; all p values > .05). Figure 11 illustrates these results. The largest correlation (r_(N=12)_ = -.482) was observed for BCs in the orientation task and improvement in proportion correct, but this did not approach significance (p=.112). Overall, there is no evidence for any pattern relating age or blindness to performance in our practical tasks.

Considering our virtual navigation task, for improvement in proportion of successful maze completions (Figure 12 A), there was a significant correlation with age only for SCs. For improvement in completion time (Figure 12 B), there was a significant correlation between performance and age for SCs, and also when SCs and BCs were considered together (Figure 12B). For improvement in the number of collisions, there were no correlations with age (Figure 12D).

The remaining panels (Figure 12 C, E, F) illustrate remaining significant correlations between improvement in performance and hearing measures. Specifically, participants’ improvement in completion time was correlated with their average absolute binaural differences in hearing thresholds for both SCs, and when SCs and BCs were considered together (Figure 12C). Furthermore, improvement in the number of collisions was significantly correlated with participants’ average absolute binaural differences in hearing thresholds for SCs and for the whole group (Figure 12E), and with their performance in the DFM test at 500Hz for the whole group (Figure 12F).

To determine statistically which of these relationships explains most of the variance in performance, we ran stepwise linear regression analyses. These showed that both participants’ improvement in completion time and number of collisions were significantly related to participants’ average absolute binaural differences in hearing thresholds, but none of the other variables (including blindness) contributed significantly above and beyond binaural hearing differences. Specifically, 24% of the variance in the improvement in completion time between session 1 and 20 was predicted by binaural hearing differences (F(1,22)=6.895; p=.015) and the relationship was positive so that smaller binaural differences were associated with larger reductions in completion times (standardized beta: .488, t(22)=2.626; p=.015). Furthermore, 25% of the variance in the improvement in number of collisions between session 1 and 20 was predicted by binaural hearing differences (F(1,22)=7.273; p=.013) and the relationship was positive so that smaller binaural differences were associated with larger reductions in collisions (standardized beta: .498, t(22)=2.697; p=.013).

In sum, for any of the practical tasks there is no evidence for relationships between improvement in performance and blindness or age. For some aspects of the virtual navigation task (i.e. time taken to complete and number of collisions made), there is evidence that improvement in the time taken to complete the mazes is significantly related to age, but overall performance improvements are better explained by differences in binaural hearing. Binaural differences, however, were correlated with age, so that older age was associated with larger binaural differences (r=.575; p=.003), making their effects confounded and difficult to assess separately. ”

f. Figure 12. Performance in the modified task clearly looks worse, and I am worried that statistical sig might have been broken by mixing together the spatial bisection and min angle tasks in the anova. Also, there is clear improvement in original spatial bisection, whichmight be linked to the known differences in subjects who are lind between egocentric and allocentric representations?

Following the reviewer 1 suggestion, we broke this down into the different tasks using t-tests and there was still no effect for any of the tasks. What we have done as per reviewer 2 suggestion was to remove this set of tasks from our manuscript, so that this is not a concern anymore.

2. The questionnaire - This is excellent, and honestly probably enough to hold up the paper in and of itself.
I was very impressed by the survey results (including the added data in the Supp file), but would appreciate it if authors could add the exact text of the questions (currently it only says Q1, Q2 etc. in the excel file), and if they could clarify if the missing phrasing asked subjects to detail their experience also when they answered No, or only when answering yes? I ask since the authors report the good feedback form the majority of subjects, but there were several subjects who did not agree with the statements but no information was supplied about what these subjects thought or felt.
I recommend that in future studies the authors use standardized quality of life questionnaires before and after so we could also see this better quantitatively.

We have added the questions to the excel file. Previously they were only contained in the manuscript, and we apologize for that.

The phrasing of the questionnaire was such that it asked for details if they said yes. But: participants also added extra comments regardless and/or at the end of the survey, which address what the reviewer is wondering about. For example, they said that they felt that their independence did not improve because they were already independent etc. What we have done is that we have added these responses into the excel file as additional items labelled to clarify that these are additional responses from participants, and as appropriate we have added to the manuscript as well.

3. Results:
Following these comments, here are the implications for the results. The authors wanted to show the following results:
Blind users were able to learn click echolocation with their training paradigm and get better at tasks - this is well supported.
Sighted users can do the same - this too is well supported
Blind users report significant quality of life improvements after 3 months - this is also well supported and a wonderful result.

Thank you.

People who were sighted learned just as well as people who were blind - I do not think the data supports this. The stats need to be corrected and verified (My current conclusion from the figures is that the current data would seem to suggest that sighted people actually did better on most tasks, but the significance might be borderline)

We have redone the statistical analysis and modified the conclusions as appropriate.

Here is the most relevant section in the discussion section:

Page 40 (line 894)

“For our practical echo training tasks, there was a noticeable trend that SCs performed overall better than BCs throughout these tasks, and we did find that SCs performed significantly better than BCs in the final session of the size discrimination task. For our virtual navigation task, we also found that SCs performed significantly better on some measures, and this was more evident for the later sessions in which error timeouts and random starts were introduced. Our secondary analyses on the nature of these group differences shed some light on the possible underlying causes. Specifically, analysis of covariance suggested that these group differences could possibly be explained based on participants’ age – our SCs were, on average, younger than our BCs. This is consistent with evidence that younger adults learn a computer-based navigation task using a sensory substitution device better than older adults [35], possibly due to experience with computer game-like tasks (e.g. for review see [42]).

Importantly, when we quantified the degree to which participants improved from session 1 to session 20 in their abilities across each of the tasks, there was no evidence for an association between age and performance in the practical tasks, suggesting that age is very unlikely to be a limiting factor in learning these echolocation abilities. We did find, however, that younger age was associated with greater improvements in the time it took people to navigate virtual mazes. It is not possible to identify a single specific perceptual or cognitive factor that might underlie this association, but we did find that binaural differences in hearing explained more variance in learning than age did. Specifically, participants with smaller binaural hearing differences improved most in training^[1]^. This is consistent with a previous report showing that people with better binaural hearing performed better in a computer-based echo-detection task [41], suggesting that differences in binaural hearing might be a limiting factor for participants learning echolocation skills through computer-based training.”

There was no difference between these groups and echolocators - I do not think the data supports this. The stats need to be corrected and verified (My current conclusion from the figures is that the current data would seem to suggest that the experts were better), but in some cases I am not sure we can legitimately compare them directly given the differences in paradigm (distance).

We have redone the statistical analysis and modified the conclusions as appropriate (and described previously here). For the distances we have removed statistical tests comparing experts and trained participants.

Here is the most relevant section of the discussion:

Page 37 (line 877):

“Overall, participants showed clear improvements in echolocation ability across a range of practical and virtual tasks, and this was the case for SCs and BCs. Specifically, in the task of size discrimination, both BCs and SCs improved considerably with training, although they did not quite reach the performance of EEs. In the task of orientation perception, BCs and SCs again improved considerably with training and, in fact, their performance at the end of training matched that of EEs. In the virtual maze navigation task, BCs and SCs improved considerably on all three measures – completion time, number of collisions, and proportion of mazes successful navigated – implying that participants did not sacrifice accuracy for speed (or *vice versa*) as training progressed. Furthermore, in terms of maze completion time and number of collisions, BCs and SCs performed comparatively to EEs, and in one instance even showed superior performance (SCs had lower completion times than EEs). The overall good performance of EEs on all our tasks (without training) suggests that these tasks are sensitive to click-based echolocation abilities related to echolocation expertise, and that they therefore have good ecological validity. Furthermore, the fact that SCs and BCs were able to perform comparatively to EEs on some measures at the end of training suggest that the structure and length of our training schedule were sufficient to bring about remarkable changes in participants’ echolocation abilities.”

Some minor technical points:
==================
1. Please fix the table column width to avoid text wrapping which breaks words and numbers to fragments. It makes the table very hard to understand.

We have improved this. The table is large and we apologize that the layout in our version is still not great. We hope that this would be better in the typeset version.

2. Subject age range in the sighted group is a bit misleading, please report age earlier and not just min/max, since the max is a significant outlier, and consider reporting the Median rather than the mean.

Yes, we have added this information earlier on in the revised manuscript, i.e. in the abstract. In the main manuscript we report all individual ages, but we do agree that this was too late.

In abstract:

“Blind and sighted participants of various ages (21-79 yrs; median blind: 45 yrs; median sighted: 26 yrs) trained in 20 sessions over the course of 10 weeks in various practical and virtual navigation tasks.”

In introduction:

Page 5 (line 93)

“Participants were either sighted (n=14) or blind (n=12), and of various ages (sighted: min 21 yrs, max 71 yrs, median 26 yrs; blind: min 27 yrs, max 79 yrs, median 45).”

In methods:

Page 6, line 116

“Fourteen sighted participants (SCs; 8 males, 6 females) took part (ages: 21, 21, 22, 22, 23, 24, 25, 27, 32, 35, 38, 48, 60, and 71; mean = 33.5, SD = 15.8, median = 26).”

3. Some abbreviations are not explicitly defined in the text (e.g. “EEs”, “BCs”), or are defined but are still confusing when encountered as the sole part of a heading (e.g. DCI, DFM). Please correct this.

Yes, we have corrected this to ensure that all abbreviations are properly defined from the outset.

Reviewer #2: General Comments and decision
The authors present a paper showing the effect of age and blindness on the learning of click-based echolocation throughout a 10 weeks program. The authors provide a strong message that can have a positive impact on the Orientation and Mobility practices and the life of people living with vision impairment: it seems like click-based echolocation is possible to learn and to use somewhat effectively, without depending on the age or condition of the person, while this new skill can even provide safer mobility and greater self-confidence.
However, the present manuscript could have been very interesting but fails to deliver by mixing different experiments that seem beyond the scope of their hypothesis and overall message. As a result, the authors present an unbalanced manuscript that is hard to follow because of the amount of experiments and results that may have little effect and relevance in the authors’ final arguments. Furthermore, while the study is focused on the effect of age and blindness, the authors did not control their study sample (blind participants) with age-matched sighted controls to properly evaluate the effect of their main factors of interest. I suggest the rejection of the manuscript as the authors need to eliminate irrelevant experiments described in the comments below and reinterpret results to improve the readability and potential impact of their work.

We thank the reviewer for their feedback and have taken their comments on board for our revision.

Strengths
The authors present many tests and training sessions and were rigorous in most of their analysis. Also, the authors present subjective reports from the participants which is still rare in the literature, such subjective reports are relevant for the field of rehabilitation as they can have more impact on policy makers and rehabilitation professionals.
Lines 1343-1344: This message is strong, positive and relevant for both the literature and rehabilitation practice. I recommend the authors to make significant changes to the manuscript so this message can be “felt” throughout the paper. I also feel like a paragraph on “implication for O&M practice” may be relevant in the context of this work, shall the authors consider it. I congratulate the authors for all the testing they’ve done. With enough trimming of the text and filtering of ideas, this manuscript may have a positive impact on the O&M profession and on the life of many clients living with visual impairments.

We thank the reviewer for their feedback. We have edited the final paragraph in the discussion to highlight applications for O&M. We have trimmed and streamlined, so that the positive impact on O&M profession is facilitated (hopefully).

Page 43 (line 965)

“Our study and results have implications for health and rehabilitation professionals working with people with vision impairments, in particular for people working in the field of orientation and mobility. Specifically, our survey demonstrates that all our participants who are blind experienced positive effects on their everyday life. Thus, also on a practical level our data are important because they demonstrate that training click-based echolocation is useful for people with vision loss, and that people can learn to echolocate regardless of age or visual status. Based on our data we therefore suggest that any time is a good time to start learning click-based echolocation, and that it would make good sense to provide training in this skill as part of orientation and mobility instruction to people with vision impairments. Furthermore, it would also make sense to provide information and training in click-based echolocation to people who may still have good functional vision (e.g. like the sighted people in our study), but who are expected to lose vision later in life because of progressive eye conditions. “

Limitations
The authors omit to describe both passive and active echolocation (click-based echolocation is an active form of echolocation). This omission leads to the authors misinterpreting parts of their results (i.e. size discrimination task / control task) and stating that their participants have no experience in echolocation while passive echolocation is a fundamental skill that blind individuals learn in orientation and mobility lessons. Furthermore, as another example, active echolocation is often used via sounds emitted by the long cane (tip against the ground) while click-based echolocation is often sidelined because of social stigma.

Yes. We have added clarification re active and passive echolocation and how click-based echolocation fits in. We have also clarified that the training and the results are with respect to click-based echolocation, i.e. people who took part had no experience in click-based echolocation prior to training, and any effects we observe are on top of people’s existing O&M skills, which may well include passive echolocation and using echoic cues from the cane.

Page 3 (line 97)

“A distinction can be made between passive and active echolocation. For passive echolocation, one listens to emissions and echoes where emissions have been made by sources other than oneself, e.g., ambient sound fields, or another person speaking, making mouth-clicks etc. For active echolocation, one makes their own emissions and uses echoes arising from those, e.g., echoes from one’s own mouth clicks, footsteps, cane taps, etc. Laboratory research has shown that click-based echolocation provides sensory advantages above and beyond passive echolocation via ambient sound fields, e.g. [18, 19, 20] or active echolocation using footsteps or cane-taps [21, 22].”

Page 42 (line 942)

“We had also asked participants about their echolocation use outside the lab, and all our participants who are blind did report in a follow-up survey that the training improved their mobility. 83% also reported that it positively affected their independence and wellbeing. All of our participants who were blind were competent long cane users and/or guide dog users prior to taking part in our training. Thus, everyone had previously already received training in orientation and mobility, and the effects that they experienced were on top of their already existing skills.”

Line 58. The argument that spatial hearing is affected by echolocation seems beyond the scope of the present manuscript. One expects the study to focus on the effect of age and blindness on the learning process of echolocation. By adding experiments on spatial hearing (i.e. spatial bisection), it makes the paper long and tedious to follow (as reflected in the total line count: Introduction line count: 56; Methods line count: 392; Results line count: 760; Discussion line count: 94). However, since the investigation of spatial hearing in echolocation is relevant for the literature, the authors could discuss their results on the matter in an additional paper.

We have followed the reviewer’s suggestion and removed these aspects of the manuscript.

Lines 106-107: The authors did not properly control their research sample (blind group) with age-matched sighted controls. As this is a study on the effect of age and blindness on the learning of active click-based echolocation, one expects the authors to recruit sighted controls that are in the same range of age (+/- 5 years of age) to properly and clearly study the effect of each factors. In the present state of the manuscript, the confusion between the age factor and the blindness factor challenges the authors’ conclusion. As this is a very positive conclusion, I would suggest the authors to control their blind group for age, which would leave a greater impact on the reader, policy makers and professionals.

At this point we cannot re-run the study due to practical and budgetary constraints. Whilst samples are similar in terms of their age range, the average age differs. In our method section we state that the reason for the age difference between samples is that it was difficult to recruit participants because of the time commitment required. Addressing this concern raised by reviewer 2, and also following suggestions by reviewer 1, we have improved our statistical analyses to support our conclusions (please see various parts of the Results section in the main text), and where appropriate we have modified our conclusions. We are pleased that our conclusion that blind and sighted people learn echolocation, and that the role played by age is limited, are supported.

Here is the most relevant section in the discussion section:

Page 40 (line 894)

“For our practical echo training tasks, there was a noticeable trend that SCs performed overall better than BCs throughout these tasks, and we did find that SCs performed significantly better than BCs in the final session of the size discrimination task. For our virtual navigation task, we also found that SCs performed significantly better on some measures, and this was more evident for the later sessions in which error timeouts and random starts were introduced. Our secondary analyses on the nature of these group differences shed some light on the possible underlying causes. Specifically, analysis of covariance suggested that these group differences could possibly be explained based on participants’ age – our SCs were, on average, younger than our BCs. This is consistent with evidence that younger adults learn a computer-based navigation task using a sensory substitution device better than older adults [35], possibly due to experience with computer game-like tasks (e.g. for review see [42]).

Importantly, when we quantified the degree to which participants improved from session 1 to session 20 in their abilities across each of the tasks, there was no evidence for an association between age and performance in the practical tasks, suggesting that age is very unlikely to be a limiting factor in learning these echolocation abilities. We did find, however, that younger age was associated with greater improvements in the time it took people to navigate virtual mazes. It is not possible to identify a single specific perceptual or cognitive factor that might underlie this association, but we did find that binaural differences in hearing explained more variance in learning than age did. Specifically, participants with smaller binaural hearing differences improved most in training^[2]^. This is consistent with a previous report showing that people with better binaural hearing performed better in a computer-based echo-detection task [41], suggesting that differences in binaural hearing might be a limiting factor for participants learning echolocation skills through computer-based training.”

With this high number of experiments, the take-away message of the authors’ work is hard to grasp. I suggest the authors to clarify the goal of each experiment and to clearly synthetize their methods and results section to improve the readers’ experience.

We have followed the suggestions of reviewer 2 and streamlined our manuscript. The discussion section is now more clearly organised around separate points that we wish to make (e.g. comparison between participants and experts; the role of age/hearing; differences between blind and sighted participants etc.). The abstract is also now more focussed and hopefully clearer

Abstract

“Understanding the factors that determine if a person can successfully learn a novel sensory skill is essential for understanding how the brain adapts to change, and for providing rehabilitative support for people with sensory loss. We report a training study investigating the effects of blindness and age on the learning of a complex auditory skill: click-based echolocation. Blind and sighted participants of various ages (21-79 yrs; median blind: 45 yrs; median sighted: 26 yrs) trained in 20 sessions over the course of 10 weeks in various practical and virtual navigation tasks. We found that both sighted and blind people improved considerably on all measures, and in some cases performed comparatively to expert echolocators at the end of training. Somewhat surprisingly, sighted people performed better than those who were blind in some cases, although our analyses suggest that this might be better explained by the younger age (or superior binaural hearing) of the sighted group. Importantly, however, neither age nor blindness was a limiting factor in participants’ rate of learning (i.e. their difference in performance from the first to the final session) or in their ability to apply their echolocation skills to novel, untrained tasks. Furthermore, in a 3-month follow up survey assessing the effects of the training on daily life, all participants who were blind reported improved mobility, and 83% reported better independence and wellbeing. Overall, our results suggest that the ability to learn click-based echolocation is not strongly limited by age or level of vision. This has positive implications for the rehabilitation of people with vision loss or in the early stages of progressive vision loss.”

Minor comments

Introduction
Line 37-38 : These statements need reference.

We have decided to remove this sentence since it does not add to the argument we are making.

Line 45 : The authors provide no example of anatomical/structural changes (rewiring or atrophy) and base this statement upon behavioral differences that may result from such neuroplastic changes. I suggest the authors to provide examples of neuroplastic changes or reformulate.

We have rephrased to make the line of argument clearer. In the beginning of this paragraph we cite reviews about neuroplastic change in response to vision loss. These reviews cover behaviour, neuroimaging, and neurophysiology. The further paragraph then focuses on neuroplastic changes as reported with respect to spatial hearing behaviour.

Page 3 (line 41)

“There is a substantial body of research investigating how the brain adapts in the context of visual sensory deprivation (for reviews see [1-8]). With respect to behavioural performance in spatial hearing tasks, it has been found that people who are blind show improved localization of sounds in the periphery [9], improved spatial tuning in the periphery [10], improved monoaural localization [11], and improved discrimination of distances of sound sources [12,9]. On the other hand, people who have been blind from an early age may struggle more when localizing the vertical location of sounds [13], or judging relative spatial position between three successive sounds [14]. In sum, visual deprivation is associated with complex neuroplastic changes with respect to spatial hearing performance.”

Lines 62-64: I suggest the authors to provide example(s) of visual-to-auditory sensory substitution. As this is a paper on audition, the example of a tactile device (without any auditory signal) to support the hypothesis of “neuroplastic changes in favor of audition” carries less weight.

This paragraph focuses on previous training work in blind and sighted people. The study we are citing is (to the best of our knowledge) the only published study systematically training blind and sighted people. Other reports are limited to training only sighted people, or there is no systematic training (i.e. only group comparison between blind and sighted are reported, but no systematic training). We have rephrased to make our argument clearer.

Page 4 (line 69)

“From a basic science perspective, investigating how people learn to echolocate and how this is related to vision loss and other aspects of their hearing would provide information on how the brain adapts in response to sensory deprivation and to learning a new skill. This more general question was addressed in one previous training study in which sighted and blind people used a visual-to-tactile sensory substitution device. It was found that after 4 sessions, blind people performed better than sighted controls [30], suggesting that visual deprivation may put people at an advantage for learning a new sensory skill. Importantly, if this is a general principle, we might expect that it would also apply to learning a complex auditory skill such as click-based echolocation, so that people who are blind would learn better than those who are sighted.”

Lines 76-77: If navigation in virtual environments can also be influenced by motor abilities, decline of cognitive abilities, auditory functions and experience with similar technology, I suggest the authors to provide reference.

The argument in this paragraph is focused on any previous work that would have investigated how older and younger people may learn novel sensory skills, e.g. sensory substitution. Whilst the variables mentioned by the reviewer are also important factors to investigate, it is beyond the scope of our manuscript. We have rephrased to clarify our argument.

Page 4 (line 78)

“From an applied science perspective, click-based echolocation may offer behavioural benefits for people who are blind, but previous reports on this have been limited to a correlational approach [31]. Thus, knowing more about learning this skill and knowing more about how it might transfer to everyday life in people who are blind is important for instruction. Furthermore, in an increasingly ageing population, age-related vision loss affects more people now than ever before and, in fact, about 80% of people with vision loss are 50 years or older [32]. Whilst the older human brain might nonetheless adapt to new challenges, the neuroplastic processes involved may differ between older and younger people [33, 34]. Yet, to date there has only been limited work investigating the learning of novel sensory skills in older age groups. Specifically, there is some evidence that, when learning to navigate a virtual environment using a sensory substitution device, older people do not learn as well as younger people do [35], suggesting merit of further investigation of the effect of age in research on neuroplasticity.”

Methods
The goal of each experiment should be made clear to help the reader understand the purpose of the study.

We have added descriptions as appropriate to improve readability.

e.g, page 13 (line 245) – orientation task

“People with expertise in click-based echolocation can use echoes to determine the contour shape of sound-reflecting surfaces, including the orientation (vertical, horizontal) of an elongated rectangle, whilst people who are new to echolocation struggle with this [23]. The orientation perception task was designed to determine how people improve in this skill through training.”

Line 105. The age difference between the groups is too large to allow a good comparison (as reflected by the authors in the discussion).

We have included additional analyses to further support our conclusions regarding age.

We have now included analyses for age for each group also and show correlations for each group in each scatterplot. For active tasks there are no significant correlations also when these are broken down by group. For the computer based tasks it seems that correlations are generally stronger for the sighted group, but the sign of the relationship is the same in the two groups.

Page 32 (line 685):

“For practical size and orientation tasks, participant improvement was not associated with age or blindness or with any of our hearing measures (max. r value = .373 for the whole group and max r value = -.482 for separate group analyses; all p values > .05). Figure 11 illustrates these results. The largest correlation (r_(N=12)_ = -.482) was observed for BCs in the orientation task and improvement in proportion correct, but this did not approach significance (p=.112). Overall, there is no evidence for any pattern relating age or blindness to performance in our practical tasks.

Considering our virtual navigation task, for improvement in proportion of successful maze completions (Figure 12 A), there was a significant correlation with age only for SCs. For improvement in completion time (Figure 12 B), there was a significant correlation between performance and age for SCs, and also when SCs and BCs were considered together (Figure 12B). For improvement in the number of collisions, there were no correlations with age (Figure 12D).

The remaining panels (Figure 12 C, E, F) illustrate remaining significant correlations between improvement in performance and hearing measures. Specifically, participants’ improvement in completion time was correlated with their average absolute binaural differences in hearing thresholds for both SCs, and when SCs and BCs were considered together (Figure 12C). Furthermore, improvement in the number of collisions was significantly correlated with participants’ average absolute binaural differences in hearing thresholds for SCs and for the whole group (Figure 12E), and with their performance in the DFM test at 500Hz for the whole group (Figure 12F).

To determine statistically which of these relationships explains most of the variance in performance, we ran stepwise linear regression analyses. These showed that both participants’ improvement in completion time and number of collisions were significantly related to participants’ average absolute binaural differences in hearing thresholds, but none of the other variables (including blindness) contributed significantly above and beyond binaural hearing differences. Specifically, 24% of the variance in the improvement in completion time between session 1 and 20 was predicted by binaural hearing differences (F(1,22)=6.895; p=.015) and the relationship was positive so that smaller binaural differences were associated with larger reductions in completion times (standardized beta: .488, t(22)=2.626; p=.015). Furthermore, 25% of the variance in the improvement in number of collisions between session 1 and 20 was predicted by binaural hearing differences (F(1,22)=7.273; p=.013) and the relationship was positive so that smaller binaural differences were associated with larger reductions in collisions (standardized beta: .498, t(22)=2.697; p=.013).

In sum, for any of the practical tasks there is no evidence for relationships between improvement in performance and blindness or age. For some aspects of the virtual navigation task (i.e. time taken to complete and number of collisions made), there is evidence that improvement in the time taken to complete the mazes is significantly related to age, but overall performance improvements are better explained by differences in binaural hearing. Binaural differences, however, were correlated with age, so that older age was associated with larger binaural differences (r=.575; p=.003), making their effects confounded and difficult to assess separately. ”

Also, the blind group is too heterogenous in age, condition and blindness onset. Since it is widely known that the onset and duration of blindness greatly influence cross-modal plasticity and behavior (reviewed in https://doi.org/10.1016/j.neubiorev.2013.08.001), I suggest the authors should divide the blind individuals into early acquired and late acquired blindness, as this might be interesting for the literature on blindness and plasticity.

We agree that this is a highly relevant question. In our sample all control participants who were blind had cause of vision loss present from birth. All were diagnosed as legally blind in childhood, with only two official diagnoses at an age that might have coincided with onset of puberty, or may have been after onset of puberty (i.e. 13yrs and 10 yrs (BC6 and BC2), but again with vision impairment having been present from birth. Thus, the majority of our participants are classified as early blind. Importantly, every blind control participant improved with training. This was also the case for the sighted group, of course. We have added clarification about our participants to the manuscript and to the discussion about late/early onset blindness and how this might affect learning and that this might be a question to pursue in future research.

Page 6 (line 118)

“Twelve blind participants (BCs; 6 males, 6 females) with no prior experience in click-based echolocation took part (details shown in Table 1). In our sample, all BCs had cause of vision loss present from birth. All were diagnosed as legally blind in childhood, with only two official diagnoses at an age that might have coincided with onset of puberty, or may have been after onset of puberty (i.e. 13 yrs and 10 yrs; BC6 and BC2), but again with vision impairment having been present from birth. Thus, the majority of our participants are classified as early blind.”

Page 42 (line 930)

“Our findings that long term visual sensory deprivation in our BCs did not put them at an advantage for learning click-based echolocation (if anything, our SCs performed better) was surprising. This is because of the large literature suggesting increased neuroplasticity in the context of visual sensory deprivation [1-8], based on which we might have expected BCs to perform better. All participants who were blind had vision loss present from birth, even though two of them received their official diagnosis at an age that might be close to or beyond the onset of puberty. Thus, the majority of our participants would be classified as early blind. There is evidence to suggest that early and late onset blindness affect neuroplastic changes [8], so that future research should investigate how the results we found here might be affected by age at onset of blindness. Based on our findings that sighted participants learned well, we would expect that people with late onset of blindness would learn well also.”

Lines 106-107: The authors show the age of all control participants, there is great difference between this data and the data from the blind participants. This leads to the confusion between the “age” and “group” factors in subsequent sections (results and discussion).

Yes, please see also our previous (and subsequent) responses regarding age.

Line 121-126: I suggest the authors to reformat their tables and add descriptive legends to improve the readers’ comprehension. 2

Yes, reviewer 1 also commented on this and we have improved this. Since tables are large they are still not great but we hope that they will be better once typeset.

Line 121-126: The authors do not provide a clear understanding about the diagnostic of some participants, example: “Tunnel vision and decreased acuity in both eyes.” For participants with some residual vision, the authors should provide visual field and acuity. Also, to be considered blind, is their residual vision low enough to be classified as “legally blind” as stated by the WHO?

Yes, all participants are legally blind. In our sample all control participants who were blind had cause of vision loss present from birth. All were diagnosed as legally blind in childhood, with only two official diagnoses at an age that might have coincided with onset of puberty, or may have been after onset of puberty (i.e. 13yrs and 10 yrs (BC6 and BC2), but again with vision impairment having been present from birth. We have added details to the table and elsewhere in the manuscript to clarify (see responses to previous comment on our blind participants).

Lines 121-126: The authors should provide the age of the participant when the diagnostic of “legally blind” was given: some participants have lost their vision later in life and some had gradual visual loss.

We have added these details to the best of our abilities. Not all participants remember the exact age when they were diagnosed. See also our previous response.

Lines 140 – 144 : Information about the information session would be relevant for getting ethics approval, however not in the context of this manuscript. I suggest the authors to synthetize as follow: “all participants were introduced to the tasks and mouth clicks prior to the first session”.

We thank the reviewer for their suggestion and have rephrased accordingly (page 9, line 158)

Lines 140 – 153: I suggest the author to add a schematic figure that represents the course of a typical session as it is not yet clear in what order tasks were completed. I feel like such a figure would greatly improve the quality of this manuscript and the understanding of the training program they put forward.

One goal for relative order of tasks was that we wanted to avoid order effects, and we also wanted to make sure that we worked in a participant-led way. Thus, for practical reasons the outdoor task was always done either in the beginning or the end of a session (in about equal parts to avoid order effects). The other, lab based, tasks were administered in different orders across participants, and to facilitate participant satisfaction we asked participants in the beginning of each session if they had a preference for the order, and if they had a preference we would just go with their preference. Thus, due to the heterogeneity of the order in which tasks were conducted a systematic figure will not be useful. We have added detail to the manuscript to make this aspect of our procedure clear.

Page 10 (line 158)

“Training took part over 20 sessions on 20 separate days spread over a 10-week period. During each training session participants performed a size discrimination task, an orientation perception task, and a virtual navigation task – all inside the lab. Either in the beginning or at the end of the session they also used mouth clicks to navigate outside the lab in natural indoor or outdoor settings for 30 minutes. Tasks were done in different orders across participants and sessions. For practical reasons, the natural navigation task was always done either in the beginning or the end of a session (in approximately equal parts). The other, lab-based, tasks were run in different orders across participants, with participants being able to choose the order if they wished. The rationale for this approach was that we wanted to avoid order effects, whilst also working in a participant-led way by maintaining participants motivation across a demanding training schedule. All tasks were always done under supervision of an experimenter. In addition to the echolocation training sessions there were two sessions during which we performed a number of basic hearing tests. Any session lasted between 2 and 3 hours, depending on how many and how long breaks participants wished to take.”

Lines 180 – 182: I suggest the authors to revise their manuscript for inconsistent paragraph spacing throughout the manuscript.

Yes, we have done this.

Line 200. I suggest the authors to reduce the sections’ heading numbering in the methods and results section. The reader have some difficulty to navigate the manuscript and to find some methods and results discussed later in the manuscript, even after some readthrough.

Yes. We have also removed sections as per reviewer 2 suggestion which has improved readability.

Line 317. The reviewer is concerned for the rights on Figure 3 since it has already been published in <https://doi.org/10.1016/j.cognition.2020.104185>.

We agree that there is similarity. The figure is different however, because it integrates different elements, and all mazes are displayed and the arrangement differs as well. To further reduce similarity we have removed the shading.

Lines 301-306: The reviewer strongly doubts that mirrored version of simple mazes can be qualified as “new” spaces, considering the number of versions (3 different mazes) and their simplicity (T vs U vs Z). The reviewers fear a lack of overall difference and novelty that prevents the authors to clearly show a generalization effect.

We have used these set of mazes since they have been used by other labs successfully for virtual navigation tasks using non-visual cues (e.g. Levy-Tzedek et al., 2016). It is true that visually these spaces may appear similar, possibly even easy. But as performance in our study shows, this is a different story when people used echo-acoustic cues in our paradigm. Most importantly, mirror symmetric (untrained) versions contain turns in opposite (i.e. mirror-symmetric) directions as compared to trained versions. As such, it does allow us to test if people just acquired some ‘rote’ responses, or if they actually understand the spatial information contained in echo-acoustic cues. If they performed by ‘rote’ they would either not be able to complete mirror-symmetric versions of previously learned mazes (i.e. they would be stuck), or their performance should (at best) match people’s performance in their first session of training. Yet, it is very clear that performance for untrained mazes is just as good as performance for trained mazes in the last sessions. This clearly demonstrates that people use echo-acoustic cues efficiently, and that they can apply them to successfully navigate a space that they have not encountered before.

We have added explanations to clarify this issue.

Page 14/15, line 278-285:

“This task is a replication of the task introduced by [28] and details are described in that report. Briefly, binaural recordings of clicks and click-echoes were made with an anthropometric manikin in physical spaces comprising corridors in specific spatial arrangements (T-mazes, U-mazes, Z-mazes) that have been used previously by other labs to investigate navigation based on non-visual cues, e.g. [35]. Whilst visually the T-, U- and Z- mazes may appear similar to one another, and possibly easy to navigate, our results suggest that this is not the case for participants navigating these mazes using echo-acoustic cues in our paradigm – that is, they had to learn how to do this. ”

Page 41 (line 921)

“Secondly, in sessions 19-20 of the virtual navigation task we measured the degree to which participants were able to navigate unfamiliar mazes (i.e. those not experienced during training). We found that participants did this very well, with no difference in performance between the unfamiliar and familiar mazes, therefore suggesting that improvements were not limited to rote-based learning, and did indeed relate to echo-acoustic skills that could be used equally well in both the unfamiliar and familiar mazes.”

Line 353. Spatial hearing seems beyond the scope of the paper. I suggest the author to discuss the experiments 2.2.3.1, 2.2.3.2, 2.2.3.3 and 2.2.3.4 and their results in a different paper that would investigate the influence of echolocation expertise in spatial hearing abilities.

We have removed these aspects of the paper.

Lines 461-463: The authors provide the subjective questionnaire and the reviewer believes that this is an additional strength for the manuscript. Did the authors consider questions assessing the degree of satisfaction/improvement on a Likert scale (auto evaluation)? This would be an interesting way to quantify their results about the real-life effect of the 10 weeks training.

We thank the reviewer for their suggestion and think that this would be interesting to apply in future work. For the current manuscript we did not use a Likert scale, but we feel that the data we collected provide a good account of people’s opinions.

Results
Lines 488 to 1248: I suggest the authors to reduce the number of figures in the results section, perhaps by making figures with multiple panels. As an example, figure 5 and 6 can be merged considering the next comments and reference: https://doi.org/10.1177/0145482X1110500103.
Lines 526-549: This experiment should not be separate from the size discrimination task. In the original work where the task was introduced, this control task is designed to control for ambient noise. This task is a passive echolocation task, the discrimination should be possible in the presence of ambient noise, as the disk can reflect or block sounds differently according to their size. In the absence of ambient sounds, this discrimination should not be possible (chance level in a 2 FCA).
Lines 547-549: This statement is false considering the true objective of this control task as it was written in <https://doi.org/10.1177/0145482X1110500103>

Yes, we agree with the reviewer that the original task was introduced as a control task for size discrimination by Teng & Whitney. In a way that is how we used it, too. For this reason, whilst providing a control for acoustic conditions like ambient noise, it is also the perfect control task to determine training effects that are not specific to click-based echolocation. For example, if people’s passive echolocation skills had improved in some way, or if they had somehow figured out a strategy to determine the location of the larger disk based on other cues not related to click-echoes, they might have improved on this task. But they did not, and this exemplifies that the benefits we observe are specific to improvement in click-based echolocation. This is not to say that the original objective of this task does not apply, but in our study it served a purpose beyond that original objective.

In response to the reviewers comment we have clarified this issue in the manuscript.

Page 12 (line 222)

“First, participants had 14 seconds to make a guess as to where the larger (reference disk) had been located. This judgment required no clicking and thus served as a control task for other training tasks. A previous study [19] originally introduced this no-click task to control for acoustic conditions like ambient noise, but here we also used it to determine training effects that are not specific to click-based echolocation. For example, if participant’s passive echolocation skills were to improve in some way, or if they were to develop a strategy to determine the location of the larger disk based on cues not related to click-echoes, they would improve on this task. Thus, any change in performance in the no-click task would indicate benefits of the training that are not specific to improvement in click-based echolocation.”

Line 552 – 557 : you report effect sizes, but what is the p value? Is it significant?

These are minimum effect sizes that we report based on our sensitivity analysis. The actual effect sizes and p-values are reported together with the other statistical results for each experiment.

Lines 584: I suggest the authors to stay consistent in the vocabulary they use, example: “proportion correct” vs “accuracy”.

Yes, thank you! We now refer to proportion correct throughout.

Lines 597-598: The authors combine the control and blind group as there is no “group difference”. This occurs multiple time in the manuscript. The reviewer believes that the groups are too heterogeneous (age, conditions) and should always remain separate for statistical analysis, even more so for the comparison with echolocation experts.

Reviewer 1 also addressed this and we have done so.

Page 22 (line 439) – size task

“Focusing only on performance in the final session, SCs performed better than BCs (t(24)=2.417; p=.024). Furthermore, EEs (n=3) performed significantly better than SCs (U=5; z=-2.042; p=.041) and BCs (U=2; z=-2.311; p=.021) did in the final session.”

Page 23 (line 480) – orientation task

“Furthermore, EEs (n=5) did not perform significantly better than either BCs (U=12; z=-1.901; p=.057) or SCs (U=21; z=-1.310; p=.190) did in the final session.”

Page 25, (line 531) – maze time

“Focusing only on completion time in session 14, there was no significant difference between SCs and BCs (t(24)=1.502; p=.146). Furthermore, BCs did not significantly differ in completion time compared to EEs (n=4; U=17; z=-.849; p=.396), but SCs were significantly faster than EEs (U=9; z=-2.018; p=.044).”

Page 27 (line 576) – maze collisions

“Focussing just on number of collisions in session 14, SCs performed significantly better than BCs (t(15.836)=3.006; p=.008), consistent with the significant main effect of ‘group’. Furthermore, EEs (n=4) did not differ significantly compared to either BCs (U=11; z=-1.578; p=.115) or SCs (U=12; z=-1.701; p=.089), suggesting that participants had learned to perform like experts by session 14.”

Page 29 (line 611) – maze success

“Focusing only on performance in session 14, there was no significant difference between BCs and SCs (t(12.839)=2.207; p=.064). Focussing just on session 14, EEs (n=4) did not perform significantly differently compared to either BCs (U=22.5; z=-.187; p=.851) or SCs (U=13.5; z=-1.836; p=.066).”

Lines 606-607: As the echolocation experts were only tested at one distance (10 cm), the reviewer believes that only the performance should be compared: with training, participants performance (at their final distance) becomes equivalent to the echolocation experts’ at 10 cm.

Also based on reviewer 1 suggestions we have revised all our comparisons between trained ppts and experts.

Lines 1132 to 1248. I suggest the authors to summarize the important factors discussed by the participants instead of presenting every quote.

We would like to keep the quotes because presenting a summary will boil down to our selection and interpretation, rather than providing ‘raw’ data (i.e. the quotes themselves). Given the shortness and brevity of the quotes a summary will also not save substantially in terms of words.

Discussion
Line 1260: Given the lack of difference between the “trained” and “untrained” mazes’ design, the reviewer doubt this conclusion can be made from the results.

Please see our previous response regarding the validity of this comparison.

Lines 1261-1262. The reviewer doubts this statement to be true since the blind group is heterogenous and not all blind participants have long term visual deprivation.

All our blind control participants were visually impaired from birth and legally blind from an early age for most cases (exceptions being BC2 and 6 who were diagnosed legally blind at age 10 and 13 yrs, respectively).

. Lines 1299 to 1310. This experiment, again, seems beyond the scope of the paper.

We removed it.

Lines 1311 to 1314: See comments on size discrimination task vs control task.

See also our response there. We still feel that whilst the original task as put forward by Teng & Whitney had the purpose to control for ambient sound, the task still allows us to determine if there is any improvement not specific to click-based echolocation, so that it is a suitable control task in our study.

Lines 1330-1344: The authors present two strong and positive conclusion paragraphs. I suggest the authors to combine them to make the final take-away message clearer and stronger.

We have only partially rewritten these two final paragraphs (to improve clarity), but would like to keep them separate, as the final paragraph makes a very distinct point about the applied implications, which can be considered separately to the preceding paragraph.

Page 43 (line 957)

“In conclusion, our results show that, somewhat surprisingly, blindness and age played only minor roles in the learning of echolocation, in particular for practical tasks, and training led to remarkable behavioural changes for all participants. Whilst there was a trend for sighted participants to perform better, most noticeably in our virtual navigation task, this is possibly explained by age, and/or binaural hearing measures related to age. Importantly, virtual navigation training effects still generalized equally for both groups, suggesting that even if blindness, age and/or binaural hearing differences may affect performance and learning, these do not impair generalization of what has been learned. Further research is needed to find out about underlying mechanisms.

Our study and results have implications for health and rehabilitation professionals working with people with vision impairments, in particular for people working in the field of orientation and mobility. Specifically, our survey demonstrates that all our participants who are blind experienced positive effects on their everyday life. Thus, also on a practical level our data are important because they demonstrate that training click-based echolocation is useful for people with vision loss, and that people can learn to echolocate regardless of age or visual status. Based on our data we therefore suggest that any time is a good time to start learning click-based echolocation, and that it would make good sense to provide training in this skill as part of orientation and mobility instruction to people with vision impairments. Furthermore, it would also make sense to provide information and training in click-based echolocation to people who may still have good functional vision (e.g. like the sighted people in our study), but who are expected to lose vision later in life because of progressive eye conditions.”

1. It should be noted, however, that binaural differences in hearing and age are correlated, making it difficult to fully separate these effects.
2. It should be noted, however, that binaural differences in hearing and age are correlated, making it difficult to fully separate these effects.
